# Culturable Bacterial Microbiota of the Stomach of *Helicobacter pylori* Positive and Negative Gastric Disease Patients

**DOI:** 10.1155/2014/610421

**Published:** 2014-07-03

**Authors:** Yalda Khosravi, Yakhya Dieye, Bee Hoon Poh, Chow Goon Ng, Mun Fai Loke, Khean Lee Goh, Jamuna Vadivelu

**Affiliations:** ^1^Department of Medical Microbiology, Faculty of Medicine, University of Malaya, 50603 Kuala Lumpur, Malaysia; ^2^Vice-chancellor's Office, University of Malaya, 50603 Kuala Lumpur, Malaysia; ^3^BP Diagnostics Centre Sdn Bhd, 30250 Ipoh, Perak, Malaysia; ^4^Department of Microbiology, Yong Loo Lin School of Medicine, National University of Singapore, Singapore 117545; ^5^Department of Medicine, Faculty of Medicine, University of Malaya, 50603 Kuala Lumpur, Malaysia

## Abstract

Human stomach is the only known natural habitat of *Helicobacter pylori* (*Hp*), a major bacterial pathogen that causes different gastroduodenal diseases. Despite this, the impact of *Hp* on the diversity and the composition of the gastric microbiota has been poorly studied. In this study, we have analyzed the culturable gastric microbiota of 215 Malaysian patients, including 131 *Hp* positive and 84 *Hp* negative individuals that were affected by different gastric diseases. Non-*Hp* bacteria isolated from biopsy samples were identified by matrix assisted laser desorption ionization-time of flight mass spectrometry based biotyping and *16SrRNA* sequencing. The presence of *Hp* did not significantly modify the diversity of the gastric microbiota. However, correlation was observed between the isolation of Streptococci and peptic ulcer disease. In addition, as a first report, *Burkholderia pseudomallei* was also isolated from the gastric samples of the local population. This study suggested that there may be geographical variations in the diversity of the human gastric microbiome. Geographically linked diversity in the gastric microbiome and possible interactions between *Hp* and other bacterial species from stomach microbiota in pathogenesis are proposed for further investigations.

## 1. Introduction

A luxurious microbial flora that is important to the health and well-being of the host inhabits human gastrointestinal tract. Gut microbiota contributes to several functions including energy harvest and storage from the diet [1], development and regulation of the gut-associated mucosal immune system [2], regulation of the central nervous system [3], detoxification of xenobiotics and carcinogens, and protection against colonization by pathogens [4]. The gut microbiota is acquired early after birth and is shaped by several factors including diet [5], genetic background, and environment [6]. Its composition and complexity can be modified by physiological changes such as aging [5] and pregnancy [7]. Fluctuations of the gut microbiota can also result from antibiotic treatment, metabolic, immunological, or infectious diseases [6]. In particular, chronic infectious and noninfectious diseases can imprint long-lasting changes of the gut microbiota that greatly impact gut homeostasis and can favor the development of other diseases [4]. The analysis of the gut microbiota and its variation is emerging as a medical approach that will be used in the prevention or treatment of diseases.

*Helicobacter pylori *(*Hp*) is a major pathogen that has been associated with humans for over 60,000 years [8]. It is estimated that more than half of the world population is infected by* Hp* [9]. However,* Hp* infection remains asymptomatic in the majority of the cases. In a small proportion of individuals, the infection leads to different diseases including peptic ulcers, chronic atrophic gastritis, gastric cancers, and gut lymphoma [10, 11]. Interestingly,* Hp* is also believed to confer on its host a protection against certain diseases including allergies, inflammatory disorders [12], and tuberculosis [13].* Hp* pathogenesis and its interactions with the gut-associated mucosal immune system have been widely studied [14]. Studies of human patients and investigations in animal models including mouse, gerbil, and monkey have generated a substantial amount of knowledge on the acute phase of* Hp* infection, establishment of colonization, activation of the gut-associated mucosal immune system, and immune escape strategies that lead to chronic colonization [14]. Human stomach is the only known natural habitat for* Hp* and once established, this bacterium is usually the predominant species of the gastric microbiota [15]. However, little is known about the modification of the gastric microbiota that results from chronic* Hp* infection and the interactions of this bacterium with other members of the gastric ecosystem. Study of the gastric microbiota has been delayed by the belief, prior to* Hp* discovery, that human stomach was a hostile environment that could not support colonization by microorganisms. Few studies have analyzed the composition of the human gastric microbiota in healthy individuals and in patients suffering from different diseases. These studies used high-throughput molecular approaches including metagenomics [15–18], terminal restriction fragment length polymorphism (T-RFLP) [19], and microarray [20] that are powerful techniques that enable the capture of DNA sequences from most of the bacteria present in the stomach including both culturable and nonculturable species. However, one important limitation in these studies is the small number of human samples (4–23 individuals) making it difficult to generate statistically significant conclusions. With regard to* Hp*, even fewer studies have analyzed the impact of this bacterium on the composition of the gastric microbiota. Recently, Hu et al. used a cultivation-based approach to isolate non-*Hp* species from gastric biopsies of 103 patients infected with* Hp* and identified most of the bacteria at the species level using MALDI-TOF mass spectrometry-based biotyping [21]. This approach has the limitation of omitting nonculturable bacteria that are predominant in the stomach [22]. However, the accuracy of mass spectrometry biotyping in bacterial identification permits rapid analysis of large number of samples.

In this study, we wanted to gain further insight into the impact of* Hp* colonization on the composition and the diversity of human gastric microbiota. We used the mass spectrometry biotyping technology to identify bacteria that were cultured from stomach biopsies of 215 patients including 131* Hp*-positive and 84* Hp*-negative subjects. The patients were affected by different gastric diseases and belonged to different ethnic groups present in Malaysia. Beside the influence of* Hp* infection, the approach taken in this study has the potential to provide an insight into the contribution of other factors such as ethnicity and disease to the composition and the diversity of the gastric microbiota.

## 2. Materials and Methods

### 2.1. Study Population

Gastric biopsy samples were obtained between 2011 and 2013 from patients referred for endoscopy examination at the University Malaya Medical Center (UMMC, Kuala Lumpur, Malaysia). Biopsy samples were taken from the antrum and body of the stomach for each patient. This study was approved by the UMMC Medical Ethics Committee and written consent was obtained from patients before being included in the study.

### 2.2. Bacterial Growth and Identification

Fresh gastric biopsy tissues were homogenized and simultaneously inoculated onto nonselective and selective chocolate agar plates (supplemented with 5% horse blood and in the case of selective media antibiotics included trimethoprim (5 *μ*g/mL), vancomycin (10 *μ*g/mL), nalidixic acid (20 *μ*g/mL), and amphotericin B (5 *μ*g/mL)). All the antibiotics were from Sigma-Aldrich Corporation (St. Louis, MO, USA). The agar plates were simultaneously incubated at 37°C under humidified condition with 10% carbon dioxide and under ambient air condition. All well-isolated colonies were selected and cultured for further studies.* Hp* colonies formed after at least three days of growth on selective plates in our culture conditions and were confirmed by positivity for urease, catalase, and oxidase tests. Additionally, Gram staining and microscopic analysis was performed to verify the presence of Gram-negative spiral rod bacteria. Non-*Hp* bacterial species (hereafter termed as other bacteria) were identified using a combination of colony morphology, mass spectrometry, and* 16SrRNA* sequencing.

### 2.3. Colony Morphology

Other colonies were picked and categorized according to morphological features including size, texture, pigmentation, hemolysis, shape, appearance, margin, and elevation. At least one colony from each morphotype present on each plate was selected. The colonies were restreaked onto non-selective plates and after sufficient growth stored at −80°C in a brain heart infusion (BHI) broth (Sigma-Aldrich, USA) supplemented with 0.4% (wt/vol) yeast extract and 20% (vol/vol) glycerol.

### 2.4. Mass Spectrometry

Ethanol/formic acid extraction and mass spectrometry was performed as recommended by Bruker Daltonics GmbH (Brême, Germany). Briefly, 1 to 2 well-isolated colonies (or a few colonies in the case of a small colony size) of similar morphotype were suspended in 300 *μ*L of ultrapure (type 1) water (EMD Millipore Corporation, Billerica, MA, USA) to which 900 *μ*L of pure ethanol were added. The samples were centrifuged (13,000 ×g, 2 min.). The supernatants were decanted and the pellets were air-dried. Once dried, 50 *μ*L of 70% formic acid (Fluka Analytical, HPLC grade) and 50 *μ*L of acetonitrile (Friedemann Schmidt Chemical, HPLC grade) were added and the samples were centrifuged again. Subsequently, 1 *μ*L of the supernatant was spotted onto a MSP 96 target polished steel BC microScout Target (Bruker Daltonics GmbH) and allowed to completely air-dry before overlaid with 1 *μ*L of fresh *α*-cyano-4-hydroxy-cinnamic acid (HCCA, Bruker Daltonics GmbH) matrix solution. The samples were dried before analysis on the Microflex LRF MALDI-TOF Mass Spectrometry System equipped with a 60 Hz nitrogen laser and microScout ion source (Bruker Daltonics GmbH). The parameter settings were as follows: delay 12719 pts; ion source 20 kV; ion source 18.34 kV; lens voltage 9 kV; mass range 2–20 kDa. Spectra were obtained in the positive linear mode after 240 shots. Raw MALDI-TOF spectra were analyzed with MALDI Biotyper 3.1 (Bruker Daltonics) using the default settings. The threshold for peak acceptance was a signal-to-noise ratio of 3. Peaks with a mass-to-charge (*m*/*z*) ratio difference >250 ppm were considered to be identical. The peak lists generated were matched against the Biotyper reference library using the integrated pattern matching algorithm. Identification score criteria used based on manufacturer were as follow: a score of ≥2.000 showed the identification in species-level, a score of 1.700 to 1.999 showed identification to the genus level, and a score of <1.700 indicated an unreliable identification. Dendrograms were created using the correlation distance measure and the average linkage algorithm settings of the Biotyper software to examine the diversity and relatedness of isolates within the species based on protein fingerprinting [23].

### 2.5. *16SrRNA* Gene Sequencing

Bacterial samples unable to be resolved by Biotyper up to the species level were analysed by* 16SrRNA* gene sequencing. Genomic DNA was prepared using RTP Bacteria DNA Mini Kit (STRATEC Biomedical AG, Berlin-Buch, Germany) according to the manufacturer's recommendations. The hyper variable regions V3 and V6 of the* 16SrRNA* gene were PCR-amplified using universal primers V3f (5′-CCAGACTCCTACGGGAGGCAG-3′)/V3r (5′-CGTATTACCGCGGCTGCTG-3′) and V6f (5′-TCGATGCAACGCGAAGAA)/V6r (5′-ACATTTCACAACACGAGCTGACGA), respectively [24]. The respective 203 and 124 bp PCR products were purified using the Wizard SV Gel and PCR Clean-Up System (Promega Corporation, Madison, WI, USA) and sequences were determined using the ABI PRISM DNA sequencer (Perkin Elmer Inc., Waltham, MA, USA). Nucleotide sequences were analysed using the National Center for Biotechnology Information BLAST software (http://www.ncbi.nlm.nih.gov).

### 2.6. Statistical Analysis

Statistical analyses were performed using the IBM SPSS 22.0 software. The diversity of the non-*Hp* culturable microbiota (number of species isolated) in the different groups (*Hp*-positive versus* Hp*-negative, ethnic groups, diseases, etc.) was analysed using independent* t*-test or one-way ANOVA. The prevalence of* Hp* and non-*Hp* bacteria in the different groups was analysed using Fischer's exact chi-squared test. A *P* value ≤ 0.05 was considered as statistically significant.

## 3. Results

### 3.1. Patient Demographic and Clinical Data

We analyzed the gastric biopsy samples from 215 patients who presented for endoscopy at the University of Malaya Medical Centre. The patients consisted of 102 (47.4%) and 113 (52.6%) men and women, respectively, with a median age of 59 years (range, 14 to 85 years). The ethnic distribution of the patients corresponded to 18 Malay (8.4%), 91 Chinese (42.3%), and 92 Indian (43.8%), the three main ethnic groups in Malaysia. The remaining 14 patients (6.5%) belonged to minor ethnic groups present in the country. One hundred eighty-five subjects (87.6%) were diagnosed with nonulcer dyspepsia (NUD), 22 (10.2%) with peptic ulcer disease (PUD), and 8 (3.7%) with gastric cancer (GC) (Table 1).

### 3.2. Prevalence and Distribution of* Helicobacter pylori* in Gastric Antrum and Body

*Hp *was successfully cultured from samples of 131 (60.9%) individuals including 60.5% of the NUD (112/185), 77.3% of the PUD (17/22), and 25% (2/8) of the GC patients (Table 1). Biopsies from which* Hp* could not be cultured were further analyzed by microscopic examination after hematoxylin-eosin staining by an independent consultant histopathologist. Samples from which* Hp* failed to be cultured and that did not show the bacterium in microscopy were considered as* Hp*-negative in this study. Prevalence of* Hp* was independent of the age, gender, or ethnicity of the patients. In contrast, significant differences were noted between the three disease groups (Table 1).* Hp* presence was comparable in NUD (77.3% positive samples) and PUD (60.5% positive samples) patients while prevalence in both groups was significantly higher than in GC subjects (25% positive samples). This result is consistent with the known progressive disappearance of* Hp* from gastric tissues during carcinogenesis [25].* Hp* can colonize different regions of the stomach mainly the antrum and the corpus. We wanted to know how* Hp* was distributed between these two gastric areas. The majority (61.8%) of the* Hp*-positive patients harbored the bacteria in both the antrum and the corpus, while 26% and 11.5% were colonized only at the antrum or the body, respectively. These figures make a total of 87.8% and 73.3% of the positive subjects colonized in the antrum and the body, respectively.* Hp* distribution in the antrum and the body was independent of the age, gender, ethnicity, and disease of the patients.

### 3.3. Species Identification of Non-*Helicobacter pylori* Bacteria

To analyze the influence of* Hp* colonization on the composition and diversity of gastric microbiota, we isolated 552 colonies from the gastric biopsies and attempted to make species identification by MALDI-TOF Mass Spectrometry biotyping. Most of these colonies were successfully identified at the species level (Table 2). However, 43 colonies (from 37 patients) belonging to the genus* Streptococcus* could not be discriminated between the species* S. pneumonia*,* S. mitis*, and* S. oralis*. Sequencing of the hyper variable regions V3 and V6 of the* 16SrRNA* gene showed that the 43* Streptococcus* colonies corresponded with* S. mitis*. Additionally, we analyzed the* 16SrRNA* gene sequences of 18 colonies (from 15 patients) initially identified as* Burkholderia fongorum* by biotyping. The Brucker reference library used in this study to identify the colonies contained limited data from* Burkholderia *bacteria that could not discriminate between species of this genus [26]. The results confirmed that the colonies actually corresponded to* B. pseudomallei*. Thirty colonies (from 23 patients) were assigned by biotyping as either* E. coli* or* Shigella* species. To unequivocally identify these bacteria, we grew the cells on McConkey agar. In this medium, lactose-fermenting bacteria (e.g.,* E. coli*) form red colonies while nonlactose fermenter bacteria (*Shigella*) form white colonies. The results showed that the 30 colonies corresponded to* E. coli*. Overall, by combining biotyping,* 16SrRNA* gene sequencing, and growth onto MacConkey medium we were able to identify, at the species level, all the other microbial colonies isolated from the biopsies.

### 3.4. Predominant Non-*Helicobacter pylori* Bacteria Isolated from the Stomach of Gastric Diseases Patients

In total, 64 other microbial species representing 3 bacterial phyla were identified (Table 2). Firmicutes (270 colonies from 208 biopsies) and Proteobacteria (220 colonies from 160 biopsies) were predominant while Actinobacteria (53 colonies from 43 biopsies) was less frequent. Additionally, 9 colonies from 8 biopsies corresponded to* Candida* species (fungal phylum of Ascomycota). At the genus level, Streptococci were the most prevalent (126 positive biopsies, 58.6%) followed by* Neisseria *(44 positive biopsies),* Klebsiella* (41 positive biopsies), and Lactobacilli (41 positive biopsies) that were each isolated from ~20% of the samples.* Escherichia coli* (23 positive biopsies) and* Rothia mucilaginosa* (20 positive biopsies) were present in ~10% of the patients. The predominant species (Table 2) comprised several human oral and upper respiratory tract commensals (*Streptococcus parasanguinis*,* S. mitis*,* S. salivarius*,* Neisseria flavescens*,* N. perflava*, and* R. mucilaginosa*) and members of the gut microflora (*Lactobacillus fermentum* and* E. coli*). Interestingly a few human pathogens or opportunistic pathogens were also isolated. These included* Klebsiella pneumonia*,* Streptococcus anginosus*,* Burkholderia pseudomallei*,* Bacillus cereus*, and* Acinetobacter baumannii*.

### 3.5. *Helicobacter pylori* Colonization Did Not Significantly Affect the Diversity of the Gastric Microbiota

Up to 12 non-*Hp* bacteria could be isolated from individual biopsies (Table 3). No non-*Hp* cells could be isolated from 27.4% (59) of the patients while 1 and 2 non-*Hp* bacteria were cultured from 23.3% and 21.9% of the samples, respectively. These figures are similar to those reported from a previous study that analyzed gastric bacteria concurrent to* Hp* infection using an approach similar to the one undertaken in this study [21]. The mean number of non-*Hp* species in single individuals was 1.93 and did not significantly differ between* Hp*-positive and* Hp*-negative patients (Table 1). This observation is consistent with previous findings suggesting that, although* Hp* colonisation might modify the gastric microflora, it does not significantly affect its diversity [15]. Similarly, the number of non-*Hp* cells isolated from biopsies did not significantly differ depending on the age, disease, gender, or race of the patients (Table 1).

### 3.6. Disease and Ethnicity Have a Greater Impact Than* Helicobacter pylori* on the Composition of Gastric Microbiota

**Figure 1:**
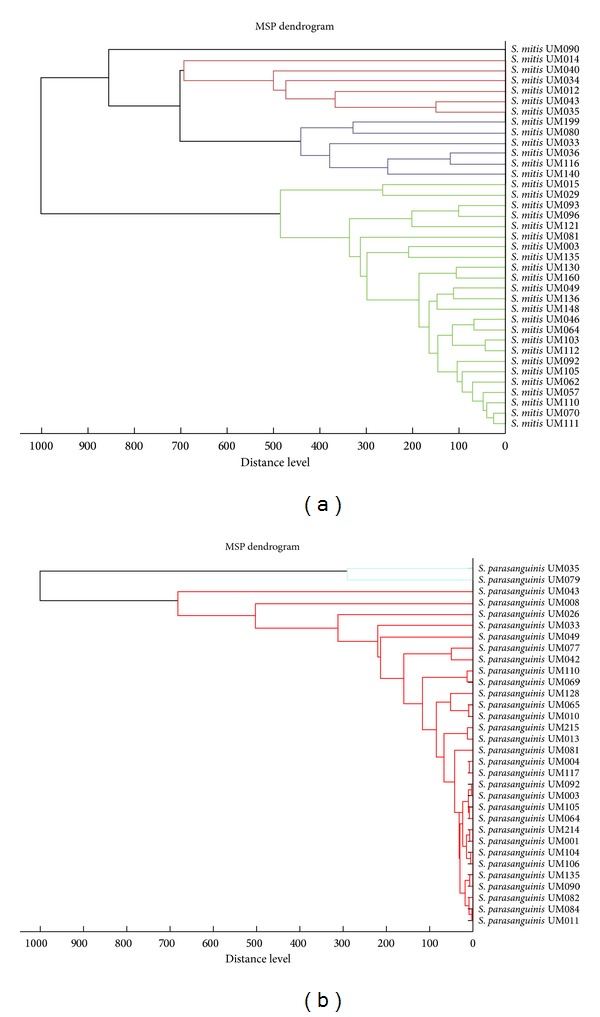
Dendrograms for clinical isolates of (a)* S. mitis* and (b)* S. parasanguinis* generated based on protein fingerprinting using Biotyper 3.1.

To analyze the influence of* Hp *on the composition of gastric microbiota in gastric disease patients, we compared the presence of individual non-*Hp* bacterial species in* Hp*-positive and* Hp*-negative subjects. Remarkably,* Bacillus cereus* was the only species that was significantly associated with the presence of* Hp* (Table 4) with all patients in which this bacterium was isolated (*n* = 10) being also colonized by* Hp. B. cereus* forms a group of versatile bacteria that can adapt to various ecological niches [27]. Human-associated* B. cereus* mainly causes food poisoning; however, we do not know what would be, if there is any, the significance of its association with* Hp* infection. Analysis of the presence of non-*Hp* bacteria in the disease groups showed that 50% and 25% of the GC patients contained* Klebsiella pneumoniae* and* Acinetobacter baumannii*, respectively (Tables 5 and 7). This prevalence was significantly higher than that in the NUD and PUD groups (Tables 5 and 7) although these results should be considered with caution because of the low number of GC compared to NUD and PUD patients. Nevertheless, they are noteworthy given the modification of the diversity and composition of the microbiota in human gastric cancer patients [19, 28]. Interestingly, chi-square analyses showed that* Klebsiella pneumoniae* and* Acinetobacter baumannii* were also differently present in the four ethnic groups (Tables 6 and 8). Both bacteria were significantly less prevalent in Chinese (*n* = 91) than in Indian (*n* = 92) (Tables 6 and 8). These two ethnic groups represented the majority of our population and were similar in size making statistical analyses reliable. None of the other bacterial species isolated in this study showed a significantly different prevalence among the four ethnic groups. Overall, these results suggest that even though* Hp* might influence the presence or absence of bacterial species, other factors have a greater impact on the composition of human gastric microbiota. Interestingly,* Streptococcus *species were isolated significantly more frequently from PUD than NUD patients (*P* value <0.005) (Table 9). Dendrograms of* S. mitis *and* S. parasanguinis* based on protein fingerprinting were illustrated in Figures 1(a) and 1(b), respectively, to demonstrate the diversity of these microorganisms isolated from the human stomach.

## 4. Discussion

In this study, we have isolated and identified bacterial species from gastric biopsies of 131* Hp*-positive and 84* Hp*-negative Malaysian residents suffering from gastric diseases and belonging to different ethnic groups. We identified 552 bacterial colonies that belonged to 61 species, 27 genera, and 3 phyla. The predominant phylum was Proteobacteria (220 colonies) followed by Firmicutes (270 colonies), while Actinobacteria were rare (53 colonies). At the species level, Streptococci were predominant (121 positive biopsies) followed by* Neisseria* (44 positive biopsies),* Klebsiella* (42 positive biopsies), and Lactobacilli (42 positive biopsies) (Table 2). Results from this study are consistent with previous studies that reported that Proteobacteria, Firmicutes, Actinobacteria, and Bacteroidetes were the predominant phyla in human gastric microbiota [15–17, 19–21]. We did not isolate Bacteroidetes since we did not use anaerobic growth conditions. We could identify up to 12 different bacterial species in individual samples. The mean number of non-*Hp* bacteria per patient was 1.93, a low number explained by the approach taken in this study that aimed to gain a qualitative rather than a quantitative insight into the composition of the gastric microbiota. Comparison of the data from* Hp*-positive versus* Hp*-negative samples suggests that* Hp* does not significantly affect neither the diversity nor the composition of human gastric microbiota. It should be noted that in contrast to previous studies, we did not use PCR amplification of genetic materials in the detection of* Hp*. Our attempt was to primarily determine whether* Hp* directly affects the diversity or composition of the gastric microbiota. Therefore, we reasoned that such an effect would require the presence of* Hp* cells in a significant number. Although this view is a hypothesis and not a demonstrated fact, we believe it to be very likely. During acute infection,* Hp* cells that arrive in the gastric lumen rapidly swim away from this harsh environment toward the epithelium [29, 30]. The spiral shape of the bacterium allows it to efficiently penetrate the loose external and the dense internal mucus layers [31, 32]. It has been shown that the majority of the* Hp* cells reside in the internal mucus layer that is firmly attached to the epithelial cells [33, 34]. This site is the natural niche of* Hp* where the bacteria efficiently proliferate as shown by many studies establishing that, when present,* Hp* is the predominant species in human gastric tissues [15, 16, 35]. Therefore, we considered as negative those samples in which this bacterium could not be neither cultured nor detected after thorough microscopic examination. The impact of* Hp* on the composition and diversity of the gastric microbiota is poorly understood. Consistent with our results, Bik et al. who used a metagenomic approach found no influence of* Hp* in the diversity gastric microbiota [15]. Similarly, Maldonado-Contreras et al. did not report a variation in the number of non-*Hp* present but found that* Hp* presence resulted in differences in the relative abundance of several phyla [20]. These observations should be considered with caution given that the mentioned studies included a small number of human subjects.

Interestingly,* Burkholderia *(15 cases),* Bacillus* (14 cases), and* Acinetobacter* (10 cases) species were isolated from the stomach of Malaysian subjects but not in similar studies on subjects from USA, China, Venezuela, Bangladesh, and Rwanda [15, 20, 21]. Melioidosis, caused by* B. pseudomallei*, is predominately a disease of tropical climates, especially in Southeast Asia and northern Australia where it is widespread. The bacterium is found in contaminated water and soil. Thus, there may be variations in the gastric microbiome between populations from different geographical regions. Goodyear et al. demonstrated in mice that* B. pseudomallei *preferentially persists in the stomach following oral inoculation and serves as a reservoir for dissemination of infection to extraintestinal sites [36]. To our knowledge, this study is the first report of* B. pseudomallei* isolation from the stomach of asymptomatic human subjects. The implication of* B. pseudomallei* presence in the human stomach deserves further investigation.

Streptococci are frequently harbored in the oral cavity. The high isolation rate (average of 56.5%) in this study suggests that these bacteria may be able to colonize the stomach rather than be just transient bacteria. Despite relative small number of peptic ulcer disease patients involved in this study, significant correlation between the isolation of streptococci and peptic ulcer disease was demonstrated. Molecular interaction between streptococci and* Hp* may play a role in ulcer disease and deserves further investigation.

Metagenomics and microarray approaches investigate into the diversity and relative quantitation of both culturable and nonculturable bacteria. In contrast, the approach adopted by Hu et al. and our study only took into account bacteria cultured under defined laboratory conditions. However, this approach was adopted because it allows further* in vitro* investigation to be carried out on non-*Hp *isolates to examine the role of coinfections by* Hp *and non-*Hp*.

*Hp* infection typically becomes chronic, induces a strong immune response, affects the production of important gastric hormones including ghrelin that acts in the central nervous system, and modifies the gastric pH [25]. Each of these effects can potentially influence the gastric microbiota. It is likely that during the evolution of* Hp* infection to chronicity, several recompositions of the gastric microbiota occur. For example, changes of gastric pH can lead to colonization by bacterial species from the oral mucosa, the upper-respiratory tract, or the intestines that cannot persist in the healthy stomach's environment. Concurrently, there might also be at different periods species that progressively disappear because the changes make the gastric milieu unfavorable for their persistence. This is exemplified by* Hp* itself that progressively disappear in gastric tissues during carcinogenesis [25]. Overall, the changes that take place in the gastric environment during* Hp* infection are complex and involve several factors with* Hp* presence being one of them. Each factor would have a minor impact and the combination of all would determine not only the composition of the gastric microbiota but also the progress toward different diseases. Consistent with this idea, Maldonado-Contreras et al. reported that the* Hp* status accounted for 28% of the total variance in the gastric microbiota of 12 individuals they analyzed [20]. Studies of large-scale human populations using throughput methods are needed to better understand the influence of the different factors that contribute to shaping of the gastric microbiota. Such studies can be validated by approaches like the one taken in this study with the ultimate goal of identifying bacterial species that could be used as microbial markers that inform about the progress toward different* Hp-*related gastric diseases.

## Figures and Tables

**Table 1:** Demographic data, prevalence of *Helicobacter pylori*, and mean number of other microbial species isolated from gastric disease patients.

	*Hp *positive N (%)	*Hp *negative N (%)	Non*-Hp*
Gender			
Male	61 (59.8)	41 (40.2)	2.13
Female	70 (61.9)	43 (38.1)	1.76
Race			
Indian	56 (60.9)	36 (39.1)	2.15
Chinese	55 (60.4)	36 (39.6)	1.6
Malay	12 (66.7)	6 (33.3)	2.28
Other	8 (57.1)	6 (42.9)	2.21
Disease			
NUD	112 (60.5)	73 (39.5)	1.9
PUD	17 (77.3)	5 (22.7)	1.68
GC	2 (25)	6 (75)	3.38

*Hp*, *Helicobacter pylori*; *N*, number of biopsies; %, percentage in the respective group; Non-*Hp*, mean number of non-*Hp* species isolated in the respective group.

**Table 2:** Other microbial species identified by Biotyper.

Microorganism	Number of positive biopsies	*H. pylori* positive *N* (%)	*H. pylori* negative *N* (%)	*P* value
*Streptococcus *	**121**	**80**	**41**	**0.091**
*S. mitis* ^†^	37	23 (62.2)	14 (37.8)	
*S. parasanguinis *	32	23 (71.9)	9 (28.1)	
*S. anginosus *	24	14 (58.3)	10 (41.7)	
*S. salivarius *	9	5 (55.6)	4 (44.4)	
*S. constellatus *	4	3 (75)	1 (25)	
*S. gallinaceus *	3	2 (66.7)	1 (33.3)	
*S. vestibularis *	3	2 (66.7)	1 (33.3)	
*S. gallolyticus *	2	1 (50)	1 (50)	
*S. gordonii *	2	2 (100)	0	
*S. peroris *	2	2 (100)	0	
*S. sanguinis *	2	2 (100)	0	
*S. australis *	1	1 (100)	0	
*Neisseria *	**44**	**33**	**33**	**0.038**
*N. flavescens *	21	16 (76.2)	5 (23.8)	
*N. perflava *	11	7 (63.6)	4 (36.4)	
*N. subflava *	7	7 (100)	0	
*N. macacae *	4	2 (50)	2 (50)	
*N. mucosa *	1	1 (100)	0	
*Klebsiella *	**42**	**23**	**19**	**0.382**
*K. pneumoniae *	41	23 (56.1)	18 (43.9)	
*K. oxytoca *	1	0	1 (100)	
*Lactobacillus *	**42**	**27**	**15**	**0.725**
*L. fermentum *	32	21 (65.6)	11 (34.4)	
*L. paracasei *	4	3 (75)	1 (25)	
*L. johnsonii *	2	1 (50)	1 (50)	
*L. reuteri *	2	1 (50)	1 (50)	
*L. salivarius *	2	1 (50)	1 (50)	
*Rothia *	**22**	**17**	**5**	**0.111**
*R. mucilaginosa *	20	16 (80)	4 (20)	
*R. dentocariosa *	2	1 (50)	1 (50)	
*Bacillus *	**14**	**12**	**2**	**0.783**
*B. cereus *	10	10 (100)	0	
*B. licheniformis *	2	1 (50)	1 (50)	
*B. firmus *	1	1 (100)	0	
*B. subtilis *	1	0	1 (100)	
*Acinetobacter *	**10**	**6**	**4**	**0.512**
*A. baumannii *	9	6 (66.7)	3 (33.3)	
*A. schindleri *	1	0	1 (100)	
*Staphylococcus *	**9**	**5**	**4**	**—**
*S. hominis *	6	2 (33.3)	4 (66.7)	
*S. aureus *	1	1 (100)	0	
*S. capitis *	1	1 (100)	0	
*S. epidermidis *	1	1 (100)	0	
*Candida *	**8**	**4**	**4**	**—**
*C. albicans *	5	2 (40)	3 (60)	
*C. glabrata *	2	2 (100)	0	
*C. tropicalis *	1	0	1 (100)	
*Actinomyces *	**5**	**4**	**1**	**—**
*A. odontolyticus *	3	2 (66.7)	1 (33.3)	
*A. graevenitzii *	1	1 (100)	0	
*A. oris *	1	1 (100)	0	
*Corynebacterium *	**5**	**5**	**0**	**—**
*C. simulans *	4	4 (100)	0	
*C. argentoratense *	1	1 (100)	0	
*Gemella *	**5**	**2**	**3**	**—**
*G. haemolysans *	2	1 (50)	1 (50)	
*G. morbillorum *	2	1 (50)	1 (50)	
*G. sanguinis *	1	0	1 (100)	
*Others *	**91**			
*Escherichia coli* ^‡^	24	15 (65.2)	8 (34.8)	0.822
*Burkholderia pseudomallei* ^†^	15	10 (66.7)	5 (33.3)	0.786
*Morganella morganii *	13	9 (64.3)	5 (35.7)	1.000
*Lysinibacillus fusiformis *	9	3 (33.3)	6 (66.7)	—
*Pseudomonas aeruginosa *	6	2 (33.3)	4 (66.7)	—
*Micrococcus luteus *	6	3 (50)	3 (50)	—
*Paenibacillus urinalis *	3	1 (33.3)	2 (66.7)	—
*Kocuria palustris *	3	1 (33.3)	2 (66.7)	—
*Enterobacter aerogenes *	3	1 (33.3)	2 (66.7)	—
*Moraxella osloensis *	2	1 (50)	1 (50)	—
*Citrobacter koseri *	2	0	2 (100)	—
*Arthrobacter castelli *	1	0	1 (100)	—
*Brevibacillus parabrevis *	1	0	1 (100)	—
*Yersinia enterocolitica *	1	0	1 (100)	—
*Granulicatella adiacens *	1	1 (100)	0	—
*Haemophilus parainfluenzae *	1	1 (100)	0	—

^†^Identified by *16SrRNA* sequencing; ^‡^lactose-fermenting organism on MacConkey agar. *P* value ≤ 0.05 is considered statistically significant. *N*, number of positive biopsies; %, percentage depending on *Hp* status.

**Table 3:** Number of other microbial species isolated from individual biopsies.

	*Hp* positive *N* (%)	*Hp* negative *N* (%)	Total
0	30 (22.9)	29 (34.5)	59 (27.4)
1	31 (23.7)	19 (22.6)	50 (23.3)
2	32 (24.4)	15 (17.9)	47 (21.9)
3	20 (15.3)	6 (7.1)	26 (12.1)
4	3 (2.3)	6 (7.1)	9 (4.2)
5	4 (3.1)	5 (6.0)	9 (4.2)
6	3 (2.3)	2 (2.4)	5 (2.3)
7	4 (3.1)	0	4 (1.9)
8	2 (1.5)	0	2 (0.9)
9	2 (1.5)	1 (1.2)	3 (1.4)
12	0	1 (1.2)	1 (0.5)

Total (*N*)	131	84	215

*N*, number of gastric samples in the respective group; %, percentage in the respective group.

**Table 4:** Prevalence of *Bacillus cereus* according to *Helicobacter pylori* status.

	*B. cereus* positive	*B. cereus* negative	Total
*Hp* positive	10	121	131
*Hp* negative	0	84	84

Total	10	205	215

**Table 5:** Prevalence of *Klebsiella pneumoniae* according to disease group.

	*Kp *pos. *N* (%)	*Kp *neg. *N* (%)	Total
NUD	35 (18.90)	150 (81.10)	185 (100)
PUD	2 (9.10)	20 (90.90)	22 (100)
GC	4 (50)	4 (50)%	8 (100)%

Total	41 (19.10)	174 (80.90)	215 (100)

*Kp*, *Klebsiella pneumonia*; pos., positive; neg., negative; *N*, number of biopsy; %, percentage in the respective group; NUD, nonulcer dyspepsia; PUD, peptic ulcer disease; GC, gastric cancer.

**Table 6:** Prevalence of *Klebsiella pneumoniae* according to ethnic group.

	*Kp* pos. *N* (%)	*Kp* neg. *N* (%)	Total
Indian	23 (25)	69 (75)	92 (100)
Chinese	9 (9.90)	82 (90.10)	91 (100)
Malay	3 (16.70)	15 (83.30)	18 (100)
Other	6 (42.90)	8 (57.10)	14 (100)

Total	41 (19.10)	174 (80.90)	215 (100)

*Kp*, *Klebsiella pneumonia*; pos., positive; neg., negative; *N*, number of biopsy; %, percentage in the respective group.

**Table 7:** Prevalence of* Acinetobacter baumannii *according to disease group.

	*Ab* pos. *N* (%)	*Ab* neg. *N* (%)	Total
NUD	6 (3.2)	179 (96.80)	185 (100)
PUD	1 (4.50)	21 (95.50)	22 (100)
GC	2 (25)	6 (75)	8 (100)

Total	9 (4.20)	206 (95.80)	215 (100)

*Ab, Acinetobacter baumannii*; pos., positive; neg., negative; *N*, number of biopsy; %, percentage in the respective group; NUD, nonulcer dyspepsia; PUD, peptic ulcer disease; GC, gastric cancer.

**Table 8:** Prevalence of *Acinetobacter baumannii* according to ethnic group.

	*Ab *pos. *N* (%)	*Ab *neg. *N* (%)	Total
Indian	5 (50.40)	87 (94.60)	92 (100)
Chinese	0 (0)	91 (100)	91 (100)
Malay	3 (16.70)	15 (83.30)	18 (100)
Other	1 (7.10)	13 (92.10)	14 (100)

Total	9 (4.20)	206 (95.80)	215 (100)

*Ab*, *Acinetobacter baumannii*; pos., positive; neg., negative; *N*, number of biopsy; %, percentage in the respective group.

**Table 9:** Prevalence of *Streptococcus* sp. according to disease group.

	*Streptococcus* pos. *N* (%)	*Streptococcus* neg. *N* (%)	Total	*P* value
NUD	98 (53)	87 (47)	185 (100)	0.003
PUD	19 (84)	3 (14)	22 (100)	

*N*, number of biopsies; %, percentage in the respective groups. *P* value ≤ 0.05 is considered statistically significant.
